# High Cervical Intrathecal Targeted Drug Delivery: A Case Report of Refractory Oropharyngeal Cancer Pain

**DOI:** 10.1155/2019/2098921

**Published:** 2019-09-10

**Authors:** Rajat N. Moman, Julie M. Rogers, Thomas P. Pittelkow

**Affiliations:** ^1^Department of Anesthesiology and Perioperative Medicine, Mayo Clinic, Rochester, MN, USA; ^2^Division of Pain Medicine, Department of Anesthesiology and Perioperative Medicine, Mayo Clinic, Rochester, MN, USA

## Abstract

**Introduction:**

Some patients with head and neck cancers have pain refractory to aggressive multimodal therapies. Herein, we report the use of an intrathecal targeted drug delivery (TDD) system catheter tip placed at C1 for the treatment of recalcitrant oropharyngeal cancer pain.

**Case Report:**

A patient with recurrent metastatic squamous cell tongue cancer reported severe pain not controlled despite high-dose opioids and nonopioid adjuvants. It was elected to proceed with an intrathecal TDD system with the catheter tip placed at the C1 level. After pump placement, we were able to decrease her daily oral morphine equivalents (OME) from nearly 1000 mg to 300 mg over the course of two months while titrating her TDD from 0.3 mg/day to 0.7 mg/day of intrathecal hydromorphone. Unfortunately, her improvement was limited secondary to aggressive cancer-directed treatments likely contributing to device infection and explant.

**Conclusions:**

In this patient, high cervical placement of an intrathecal TDD catheter was associated with a decrease in OME. While used in clinical practice on occasion, the use of high cervical TDD placement such as this implantable C1 intrathecal TDD system for cancer-associated pain is underreported in the literature. Further studies on this intervention within this challenging population are warranted.

## 1. Introduction

Cancer-associated pain is a worrisome problem and is accompanied by numerous psychosocial responses and a diminished quality of life. According to a recent meta-analysis, the prevalence of pain is 66.4% in advanced, metastatic, or terminal cancer and is 55.0% during anticancer treatment [1]. By comparison, a systematic review of pain in patients with head and neck cancer found 50% of patients had pain prior to cancer therapy, 81% had pain during cancer therapy, and 36% had pain 6 months after treatment. Furthermore, the reported intensity of posttreatment pain was often worse than pretreatment pain [2]. It appears that cancer in general, and head and neck cancers in particular, is commonly accompanied by distressing levels of pain.

Given the considerable amount of pain patients with cancer experience, some patients require treatment beyond routine systemic analgesics in the setting of tolerance or side effects with systemic opioids [3], or in the setting of intractable pain. Intrathecal delivery of opioids is an established intervention for patients with poorly treated cancer-related pain [4, 5] and has been associated with lower pain intensity scores and mean morphine equivalent doses [6–9]. The catheter tip is generally placed near to the site of greatest pain, based on the pain physician's clinical judgement. Optimal placement nearest the site of pain may be difficult in patients who experience pain in the head and neck region due to concern for side effects of intrathecal pain medications in the cervical region near critical structures of the brainstem. The literature on implanted intrathecal TDD for head and neck cancer pain focuses on catheter placement outside of the cervical region [10], and, while TDD catheter tips are placed high in the cervical spinal canal in clinical practice, there remains a paucity of literature reporting this technique and associated outcomes. Our report is on the successful use of an implantable intrathecal TDD system catheter tip placed at C1. Intrathecal delivery of analgesics can involve the use of an intrathecal catheter attached to an external infusion [6, 11] or an implanted pump. Studies of intrathecal opiates have focused on external infusions in patients who are on hospice or patients who have pain from cancer outside of the head and neck area [9]. Our case report focuses on the use of an implanted intrathecal TDD system to improve the quality of life of a patient undergoing cancer-directed treatment for a head and neck cancer who remains hopeful for a cure. Herein, we describe a case of a patient undergoing treatment for recurrent metastatic oropharyngeal squamous cell cancer who is experiencing severe, cancer-associated pain and underwent implantation of an intrathecal TDD system with catheter placement at the C1 level with significant decreases in daily oral morphine equivalents (OME).

## 2. Case Report

This is a 59-year-old woman with recurrent, metastatic squamous cell tongue cancer who is status post two partial glossectomies, right and left neck dissections, a floor of mouth resection, a neck dissection with radial forearm free flap, tracheostomy, and chemoradiation therapy. She had experienced constant facial pain about the region of the oropharynx, left tongue, and mandible of variable intensity for two years. Additionally, she had prominent nocturnal pain that would awaken her from sleep. She described her pain as throbbing, burning, and sharp in quality. Talking, mucous secretion management, and application of medications for her dental carries all exacerbated her pain. On an 11-point numeric rating scale where 0 is no pain and 10 is the maximal pain imaginable, her average pain intensity was 8/10 and was as high as 10/10. Her level of functioning had declined to the point that her oncologist was unable to offer further chemotherapy treatment. In the setting of her cancer recurrence, she unfortunately struggled with chemical coping and opioid misuse. She had a recent admission to the hospital for a pain crisis during which severe pain limited her oral intake and her ability to communicate verbally. Prior to this admission for her pain crisis, she was on a home regimen of methadone 10 mg every 6 hours, oral morphine 60 mg every 3 hours (600 OME), gabapentin 600 mg three times daily, and venlafaxine 100 mg three times daily. During her admission for a pain crisis, her OME was increased to 2000 mg daily. In addition, a ketamine infusion and lidocaine infusion were initiated to control her intractable pain. Once her pain stabilized, she was transitioned to oral medications and discharged on methadone 15 mg every 6 hours and hydromorphone solution 12 mg per gastric tube every 2 hours (900 OME). Around this time, magnetic resonance imaging of her brain was negative for leptomeningeal metastasis. After discharge, her pain remained a 7/10 and the patient elected to have an intrathecal TDD system placed.

The patient was taken to the operating room. At the time, her last white blood cell count was 6.5 × 10^9^/L. Given her complex airway, she was provided monitored anesthetic care. The patient was placed in a right lateral decubitus position. Previous reports [12] suggest a high cervical entry point; however, in order to reduce the risk of cervical spinal cord injury, a more traditional intrathecal targeted drug delivery approach was undertaken. After gaining intrathecal access at T12/L1 via a left paramedian approach, the catheter was easily advanced to the top of C1 under live fluoroscopy. Her pump was placed in the subcutaneous tissue overlying her left lower abdomen. A hydromorphone infusion was initiated at 300 mcg per day. This medication was chosen over morphine due to its slightly more hydrophobic nature, the anticipated higher flow concentration of CSF within the cervical spine, and, theoretically, a decreased side effect profile [13]. She was admitted to the intensive care unit for cardiopulmonary monitoring initially and remained stable without any event until discharge from general care two days later.

She was seen in follow-up by pain and palliative care physicians as an outpatient over the next eight weeks. During her follow-up, her intrathecal hydromorphone dose was gradually increased to 700 mcg per day by increasing her dose every several days and her systemic use of oral opiates declined to 300 OME with stable pain intensity. During this time, she endorsed being able to use much less of her breakthrough medication and spread her scheduled opioid medications from every two hours to every four hours. Indeed, her pain was so reasonably well controlled that her oncologist planned to resume her treatment. Given the patient's desire to pursue aggressive cancer-directed treatments, her cytotoxic chemotherapy and immune therapy were initiated three days post TDD system placement. Her next white blood cell count check after chemotherapy was started was 3.3 × 10^9^/L. Unfortunately, approximately two months after device implantation, she developed an infection around the reservoir pocket requiring hospitalization and TDD explant.

## 3. Discussion

This patient had severe unrelenting pain in the setting of recurrent oropharyngeal cancer, which was limiting her ability to receive desired cancer treatment and greatly impacted her quality of life. She additionally struggled with opioid misuse as a means to chemically cope with her terminal diagnosis. Despite aggressive attempts with repeated hospitalizations to treat her pain, she experienced limited effect and the patient and her care team elected to pursue an implantable intrathecal TDD system with the catheter tip placed at the C1 level. As outlined in the guidelines, the catheter tip location, patient goals and performance status, and cerebrospinal fluid dynamics are the most important characteristics to consider when placing an intrathecal catheter [4]. After placement of the intrathecal pain pump and initiation of intrathecal hydromorphone, she did decrease her OME significantly in a controlled fashion while reporting stable pain intensity. In addition to decreasing her OME, she endorsed that her quality of life had improved and she was able to resume chemotherapy shortly after implantation. Our pain assessment focused on the use of an 11-point numeric rating scale where 0 is no pain and 10 is the maximal pain imaginable because of its ease of use and understanding by patients. However, there are other tools that globally assess quality of life of patients with cancer that have been shown to be effective and have been validated for use in patients with cancer [14–16]. One such tool that focuses on the evaluation of patients with head and neck cancer is the EORTC QLQ-H&N35 evaluation that incorporates mouth pain, swallowing problems, senses, speech, social eating, and social contact, as well as many single scale items. While we did not utilize this measure in our assessment of our patient, this validated tool may have measured the improvements in her quality of life that she reported and may be valuable to include in future prospective assessments [15]. Ultimately, the therapeutic benefit of our patient's intrathecal TDD system was limited secondary to the aggressive nature of her cancer-directed treatments likely resulting in poor wound healing and ultimately infection with device explant. Despite the limited effect in the setting of infection, high cervical placement of an intrathecal pain catheter with an implanted TDD system appears to be an efficacious strategy for treatment of severe, cancer-associated pain.

## 4. Conclusions

Based on this case report, high cervical intrathecal TDD may be an effective method of treating severe, cancer-associated head and neck pain. In this case, an aggressive chemotherapy regimen may have contributed to limited therapeutic benefit for our particular patient. Experimental studies of TDD are warranted for this often challenging to treat population.
